# Composite Coatings of Chitosan and Silver Nanoparticles Obtained by Galvanic Deposition for Orthopedic Implants

**DOI:** 10.3390/polym14183915

**Published:** 2022-09-19

**Authors:** C. Zanca, S. Carbone, B. Patella, F. Lopresti, G. Aiello, V. Brucato, F. Carfì Pavia, V. La Carrubba, R. Inguanta

**Affiliations:** 1 Department of Engineering, University of Palermo, RU INSTM, Viale delle Scienze, 90133 Palermo, Italy; 2 Consorzio Universitario di Caltanissetta, Corso Vittorio Emanuele 92, 93100 Caltanissetta, Italy; 3 ATeN Center, University of Palermo, Viale delle Scienze, 90133 Palermo, Italy

**Keywords:** chitosan, Ag nanoparticles, 304L stainless steel, coating, galvanic deposition, corrosion, orthopedic implant

## Abstract

In this work, composite coatings of chitosan and silver nanoparticles were presented as an antibacterial coating for orthopedic implants. Coatings were deposited on AISI 304L using the galvanic deposition method. In galvanic deposition, the difference of the electrochemical redox potential between two metals (the substrate and a sacrificial anode) has the pivotal role in the process. In the coupling of these two metals a spontaneous redox reaction occurs and thus no external power supply is necessary. Using this process, a uniform deposition on the exposed area and a good adherence of the composite coating on the metallic substrate were achieved. Physical-chemical characterizations were carried out to evaluate morphology, chemical composition, and the presence of silver nanoparticles. These characterizations have shown the deposition of coatings with homogenous and porous surface structures with silver nanoparticles incorporated and distributed into the polymeric matrix. Corrosion tests were also carried out in a simulated body fluid at 37 °C in order to simulate the same physiological conditions. Corrosion potential and corrosion current density were obtained from the polarization curves by Tafel extrapolation. The results show an improvement in protection against corrosion phenomena compared to bare AISI 304L. Furthermore, the ability of the coating to release the Ag^+^ was evaluated in the simulated body fluid at 37 °C and it was found that the release mechanism switches from anomalous to diffusion controlled after 3 h.

## 1. Introduction

The incidence of periprosthetic tissue infections is among the most common complications in traumatological and orthopedic surgery [1,2]. Because of this, the patient undergoes prolonged antibiotic therapy and sometimes is forced to undergo multiple surgical treatments that could lead to bacterial resistance. According to some statistics, periprosthetic joint infections have an incidence of 1.3%, and 22% of patients are subjected to a second operation for complications due to infections [3,4]. Obviously, this leads to an increase (up to seven times) in the costs of hospitalization and especially to a stressful condition of the patient. For these reasons, consistent efforts to the prevention of infection are mandatory in order to ensure the overall health of the patient. Preventing bacterial colonization of the surface of the orthopedic devices is a key strategy for limiting the onset of infections [3,4,5]. In this frame, antibacterial coatings have become a very challenging field of research, strongly stimulated by the growing urgency of identifying alternatives to the traditional administration of antibiotics [6]. Among antibacterial coatings, those based on biopolymers have played a key role [7]. One of the most studied biopolymers is chitosan (CS) [8,9], a natural biopolymer, that is the second most copious polysaccharide in nature after cellulose. CS results from alkaline deacetylation of chitin [10] which is extracted mainly from the exoskeleton of crustaceans and fungi [11]. The deacetylation degree of CS is linked to its biological activity which is higher for a higher deacetylation degree [12]. CS has a pKa value of 6.5 [13], which means that the protonation of amino groups can occur in diluted acidic aqueous solutions, making it suitable for many applications such as food technology [14], agriculture [15], and water waste treatments [16]. Its main use is for biomedical applications, thanks to its biocompatibility, biodegradability, antimicrobial, and mucoadhesive ability [11]. In addition, CS has been studied for its antibacterial properties against fungi, Gram-positive, Gram-negative, and yeasts [17,18,19]. Thanks to all these properties it was used in biosensors [20,21], drug delivery [22,23], wound healing [24,25], tissue engineering [26,27,28], and bone healing [29,30].

To improve the antibacterial properties of different types of coating, in several studies the addition of silver nanoparticles (AgNPs) was proposed [31,32,33,34]. AgNPs can physically interact with the cell surface of various bacteria. The interaction changes depending on the shape and size of the nanoparticles. In particular, it was shown that smaller nanoparticles have an increased ability to penetrate the cell membrane modifying the intracellular structure [35,36,37]. In addition, AgNPs have the ability to increase the production of reactive oxygen species (ROS) and free radicals in cells [38]. The presence of H_2_O_2_ in ROS triggers the following Fenton-like reaction which leads to the formation of silver ions

(1) $$Ag+H_{2}O_{2}+H^{+}\to Ag^{+}+OH^{.}+H_{2}$$

that are highly cytotoxic to bacteria also at very low concentrations [39,40,41].

Considering the properties of CS and AgNPs, in this work a composite coating for AISI 304L steel was investigated. The traditional deposition methods of antibacterial coatings are electrophoretic deposition [42,43] and electrodeposition [44,45]. In this work to obtain the composite coating of CS/AgNPs we have used the galvanic deposition method. It is a very versatile method suitable to deposit different materials [46,47,48] and also in nanostructured form [49,50,51,52,53,54,55,56,57,58]. Its particularity consists of a spontaneous deposition that does not necessitate any external power supply. Basically, the galvanic contact between a working electrode and a sacrificial anode drives the overall process in an electrochemical cell containing a solution of the coating precursors [59]. In particular, the difference in the electrochemical redox potential of the galvanic couple plays a key role to deposit coatings on the working electrode used as cathode. The reactions that occur at the cathode depend on the electrolytes dissolved in the deposition solution. In this work, since nitrate ions were added in the deposition solution, the reactions of base electrogeneration are triggered which leads to a local pH increase in the electrode/electrolyte interface. This pH increase leads to the deposition of CS as soon as the value of pKa was reached. During the deposition the CS incorporates the silver nanoparticles thus forming the composite coating. In galvanic deposition it is very simple to tune the rate of deposition because the coating growth depends on the ratio between anodic and cathodic areas. Being a spontaneous process, it does not require sophisticated equipment or a formed operator. According to Blanda et al., CS coatings carried out by galvanic deposition were distinguished for good adhesion on the substrates and the lack of cytotoxicity [60].

The purpose of this work is the study of the performances of CS/AgNPs coating in terms of corrosion protection of the metallic substrate in body fluid since the antibacterial behavior has been widely investigated in the literature [45,61,62,63,64,65,66,67,68]. Austenitic stainless steels exhibit high corrosion resistance, and they are suitable to fabricate orthopedic devices. Nevertheless, they are subject to pitting corrosion in chloride environments. Chloride ions are the main responsible for initiation step of pitting corrosion. In addition, the lack of Mo in 304L steel makes it more exposed to the risk of chloride attack respect to AISI 316L [69,70,71]. Thus, corrosion tests were carried out in simulated body fluid (SBF) at 37 °C to simulate the human body environment in which the chloride ions are present. Physical–chemical characterizations of the coatings were carried out to investigate morphology and chemical composition. Furthermore, the ability of the coating to release Ag^+^ ions in SBF was also studied.

## 2. Materials and Methods

**Figure 1:**
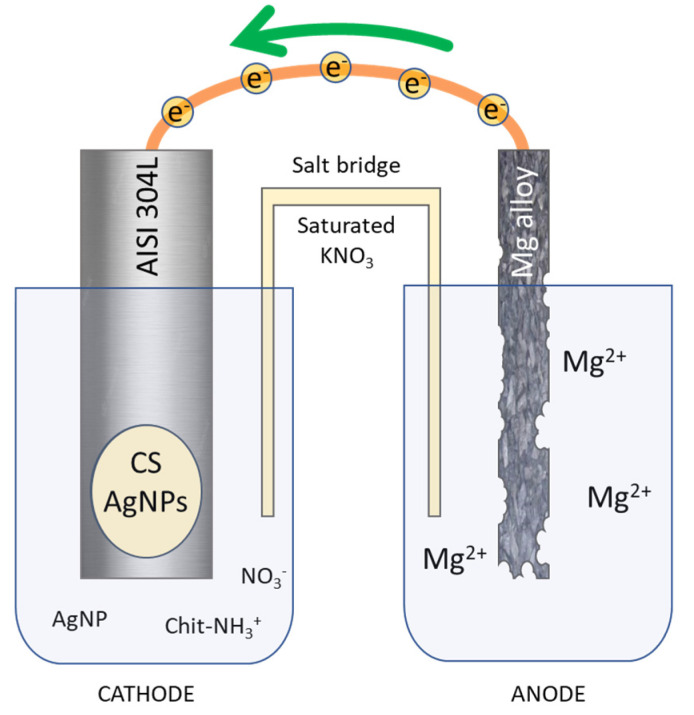
Schematic representation of galvanic deposition of chitosan/Ag nanoparticles.

Commercial AISI 304L Stainless Steel (SS) (0.022 %C, 18.13 %Cr, 8.052 %Ni, 1.208 %Mn, 0.355 %Si, 0.0165 %P, 0.0109 %S, Fe at bal.) bars (3 × 15 × 70 mm^3^) were used as substrate in order to deposit CS/AgNPs coatings. As a sacrificial anode, a sheet of Mg/Al alloy (30 × 70 × 1 mm^3^) was used. Firstly, metallic surfaces were degreased in an ultrasonic bath in pure acetone for 10 min. Afterward, mechanical polishing with abrasive papers (P150, P300, P800, P1200) was carried out. Finally, a rinsing was conducted in deionized water and acetone three times, each lasting 5 min. After degreasing process, surface was delimited with an insulator in order to expose an active area of 1.13 cm^2^ and 27 cm^2^ for cathode and anode, respectively. Galvanic deposition was conducted in a two-compartment cell (Figure 1) connected by a salt bridge filled with agar/agar in saturated KNO_3_.

The cathodic solution was prepared in different steps, in order to allow the formation of the complex between chitosan and silver ions. Initially, in 100 mL of Milli-Q water at 70 °C, 1 mL of pure acetic acid was added to promote the solubilization of chitosan thanks to the protonation of amino groups according to the reaction:

Chit− NH_2_ + H_3_O^+^ = Chit− NH_3_^+^ + OH^−^ (2)

Different CS concentration was solubilized (7.5 gL^−1^ and 10 gL^−1^) in order to study the effect of this parameter on the coating performance. These compositions were selected after a systematic investigation carried out on pure CS coating obtained by galvanic deposition [60]. Once the CS was solubilized, 0.2 M of sodium nitrate was added. When a homogeneous solution was obtained 1 mL of silver nitrate solution 0.05 M was added. During mixing, a cationic complex begins to form due to the amino groups of chitosan that are able to interact with silver ions according to the reaction reported in [61,72]:

Ag^+^ + 2Chitosan-NH_2_ → [Ag(Chitosan-NH_2_)_2_]^+^ (3)

The presence of chitosan acts as a reducing agent in order to transform silver ions into metallic silver, and as a stabilizing agent to avoid the agglomeration of AgNPs. After silver nitrate solubilization, the solution pH was adjusted at 5 with NaOH. The solution was maintained under continuous stirring for 6 h at 70 °C at a speed of 650 rpm. At the end a change in color from transparent-yellow to yellowish brown was noted, demonstrating the formation of AgNPs [73,74]. According to Yadav et al., the addition of NaOH stimulates the formation of nuclei and the growth of AgNPs [75] and promotes a hyperchromic effect for which an increase in the absorption peak of the solution is expected.

As anodic solution, sodium chloride 1 M was used. The tops of the metal sheets were short-circuited and galvanic deposition was carried out at 50 °C for 6 h. For each experiment a fresh solution was used.

The morphological features were scrutinized using a FEG-ESEM microscope (model: QUANTA 200 by FEI) equipped with an X-ray energy dispersive spectrometer (EDS) to perform chemical composition analyses. X-ray diffraction was performed to evaluate the crystallographic structure of the deposit using a RIGAKU X-ray diffractometer (model: D-MAX 25600 HK). Diffraction patterns were investigated in the 2θ range between 10° and 80° degrees with a tube voltage of 40 kV and current of 100 mA. Diffraction peaks were attributed by comparison with the literature data and considering the ICDD database [76]. These characterization methods are detailed in [77,78,79,80,81]. UV-VIS spectroscopy was used to study the formation of silver nanoparticles (model Infinite^®^ 200 PRO, TECAN).

An FT-IR/ATR instrumentation (FT-IR/NIR Spectrum 400 spectrophotometer from Perkin-Elmer Inc., Wellesley, MA, USA) was used to investigate the chemical surface composition of the materials. For each sample, 4 accumulations scans in the range of 4000–400 cm^−1^ were collected with resolution set at 4 cm^−1^.

The melting temperature of the CS-based coatings was evaluated via differential scanning calorimeter (Setaram Instrumentation, Caluire, France, model DSC131) and compared to the as-received CS powder. The analysis was carried in the temperature range of 50–175 °C at a 10 °C min^−1^ heating rate, under nitrogen flow.

The corrosion performances of the coatings were evaluated by electrochemical characterization in SBF (the preparation procedure is detailed in [82]) at 37 °C for an aging time of 21 days. Electrochemical characterizations were performed through a conventional three-electrode cell in which Pt wire and Ag/AgCl (3M KCl) were used as counter and reference, respectively [60,82,83,84]. Specifically, open circuit potential (OCP) measurements and potentiodynamic polarization were performed. From the polarization curves, the corrosion potential (E_corr_) and corrosion current density (i_corr_) were calculated through the Tafel extrapolation method. Electrochemical impedance spectroscopy (EIS) was also carried out in the frequency range between 0.1 Hz and 100 kHz, with a 0.010 V of AC perturbation. Impedance data were fitted using the ZSimpWin software.

For the evaluation of the Ag^+^ release curve, series of silver sulfadiazine (Ag-SD) solutions in SBF with Ag-SD were used to build the calibration line. Ag-SD/SBF solutions with concentrations from 6.25 to 100 mgL^−1^ were prepared and analyzed via UV–Vis (model UVPC 2401, Shimadzu Italia s.r.l., Milan, Italy) at a wavelength of 262 nm [85], using SBF as reference. In this range of concentration, the calibration curve was linear (ABS_262nm_ = 0.0017 [Ag^+^]; R^2^ = 0.999; Figure S1). Ag^+^ release was evaluated by immersing an area of 2 cm^2^ in 15 mL of SBF at 37 °C. The absorbance of the medium was measured three times at different time points using SBF as reference and the ions concentration was evaluated by using the calibration line. After each time point, the samples were dipped in 15 mL of fresh SBF stored at 37 °C. The reported graphs display the cumulative release of Ag^+^ evaluated by sequentially adding the amount of Ag^+^ released after each time point. The experiment was conducted at different time points and interrupted after 144 h.

## 3. Results and Discussion

### 3.1. Galvanic Deposition

Before proceeding with coatings deposition, the presence of AgNPs in the cathodic solution was firstly evaluated by UV-VIS spectroscopy. In particular, the spectra of two solutions containing CS and CS/AgNPs were detected and compared (Figure S2a). In the spectrum of CS/AgNPs solution (red line) it can be observed a band at 420 nm. As reported in the literature [86,87,88], this is characteristic of the surface plasmon resonance of AgNPs. On the other hand, no absorption peak was detected for CS. Thus, AgNPs were formed (see reaction 3) and CS molecules act as reducing and stabilizing agents. This is also confirmed by the color change of the solution that became yellowish brown (Figure S2b,c), typical of the formation of AgNPs. In addition, the presence of a single peak of plasmon resonance gives information about AgNPs shape. In particular, according to Mie’s theory, a single peak is typical of the spherical shape, since nanoparticles with asymmetric shapes lead to the formation of multiple resonance peaks [89,90].

The galvanic deposition was conducted in a cell schematized in Figure 1. Galvanic coupling between AISI 304L and Mg/Al alloy represents the core of the entire process. The electrodes were short-circuited and immersed in the solution, the dissolution magnesium of the sacrificial anode, immersed in NaCl solution, following the reaction occurs:

Mg→Mg^2+^ + 2e^−   ^ (E^0^ = −2.36 V/NHE) (4)

Mg/Al alloy was chosen as the sacrificial anode due to the very low redox potential of magnesium to take advantage from metal electrochemically coupled [60]. The electrons released from anodic reaction flow toward the working electrode. Once the electrons reach the AISI 304L surface, the electrogeneration of base reactions take place at the electrode/electrolyte interface. In particular the following reactions occur [91]:

NO_3_^−^ + H_2_O + 2e^−^ → NO_2_^−^ + 2OH^−^   (E^0^ = 0.0835–0.059 pH V/NHE) (5)

2H_2_O + 2e^−^ → H_2_ + 2OH^−^      (E^0^ = 0.00–0.059 pH V/NHE) (6)

O_2_ + 2H_2_O + 4e^−^→4OH^−^       (E^0^ = 1.23–0.059 pH V/NHE) (7)

The hydroxyl ions produced from these reaction causes the local increase in solution pH near the surface of the working electrode. This local increase in pH causes the deprotonation of amine groups of CS, for pH value above pKa, by the following reaction:

Chit− NH_3_^+^ + OH^−^ = Chit− NH_2_ + H_2_O (8)

Following this reaction pathway, deposition of CS occurs on the working electrode and concurrently AgNPs are trapped inside of polymeric matrix. Simultaneously with the deposition of the CS/AgNPs coating, hydrogen bubbles start to be produced at the surface of the working electrode due to reduction in water molecules (reaction 6), leading to the formation of a very porous structure. The two-cell configuration was chosen to prevent the magnesium coming from the dissolution of the anode from contaminating the coating, as already experienced in our previous work [60].

### 3.2. Characterizations

**Figure 2:**
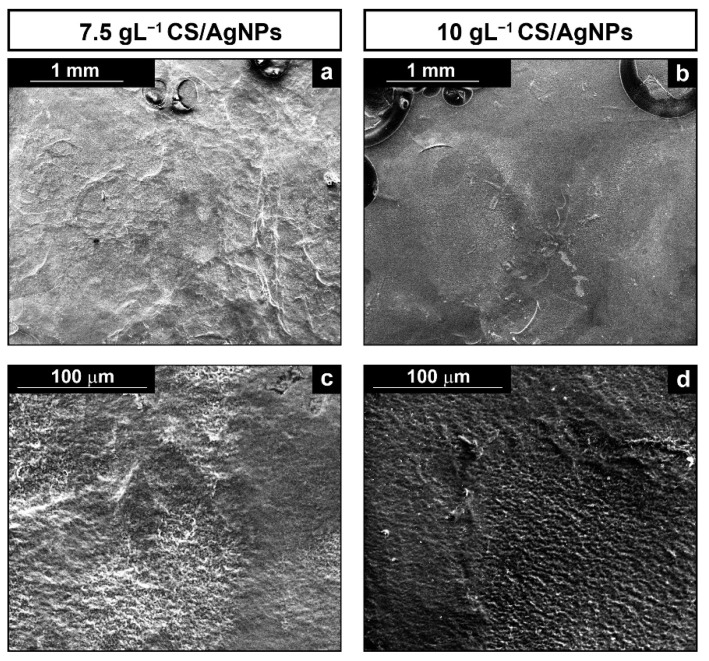
SEM images of deposits obtained using different CS concentrations: (**a**,**c**) low and high SEM magnification of the composite coating obtained using 7.5 gL^−1^ of CS solution; and (**b**,**d**) low and high SEM magnification of the composite coating obtained using 10 gL^−1^ of CS solution.

In order to evaluate the morphology of the composite coating, the samples were analyzed by SEM. Coatings were obtained through galvanic deposition in solution with different amounts of CS, Figure 2, while the concentration of AgNPs was maintained constant, with 1 mL of 0.05 M AgNO_3_ solution as mentioned before.

**Figure 3:**
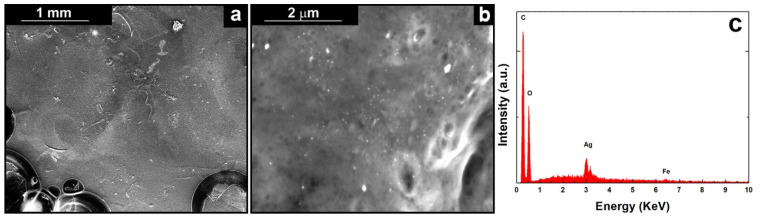
(**a**) Low, (**b**) high magnification SEM images and (**c**) relative EDS spectrum of the coating obtained using a solution of 10gL^−1^ of CS.

In Figure 2, it can be observed the presence of homogeneous coatings that cover the metallic substrate regardless of CS concentration in the solution. Also, circular macropores surrounded by a compact film are present, as better evident in the upper and lower area of Figure 2b and Figure 3a, respectively. According to [42,92,93], this feature is due to the deposition of CS in combination with hydrogen bubbles generation during galvanic process. In particular, as reported by Mąkiewicz et al., the high viscosity of the deposition solution facilitates the process of adhesion of gas bubbles on the surface of the cathode [94]. Gas bubbles create a barrier at the interface between electrode and electrolyte that hindered the deposition of the coating in that area. When the H_2_ bubbles exceeds a certain critical size, they detach from the electrode and the CS deposition can resume. The final effect is the formation of a porous coating as the bubbles act as a dynamic template [95,96]. As reported in [97], the formation of a porous coating might be a plus point since it could promote cells growth.

Figure 3b shows a high magnification image of the coating obtained using a solution containing 10gL^−1^ of CS, where the Ag nanoparticles that are incorporated in the polymeric matrix can be observed. Further qualitative information comes from EDS analysis where Ag peaks attest the presence of AgNPs, Figure 3c. Carbon and oxygen peaks belong to hydrocarbon chains of CS. The weak peak of iron, belonging to AISI 304L, is due to the presence of the coating that shields the signal. After 21 days of aging in SBF, the morphology (Figure S3a) is not characterized by substantial differences compared to as-prepared coatings. Thus, it can be concluded that the composite coatings are stable in the physiological conditions. However, the presence of some crystals on the surface can be noted, probably due to the precipitation of some components of SBF after dehydration of the coating prior SEM analysis. This is confirmed by EDS analyses that showed the presence of new elements such as calcium, phosphorous, chlorine, sodium, and sulphur present in the SBF solution (Figure S2b). It is important to highlight that the silver peak has a very low intensity after aging. This is a good result because means that the AgNPs have been correctly released during aging in SBF.

**Figure 4:**
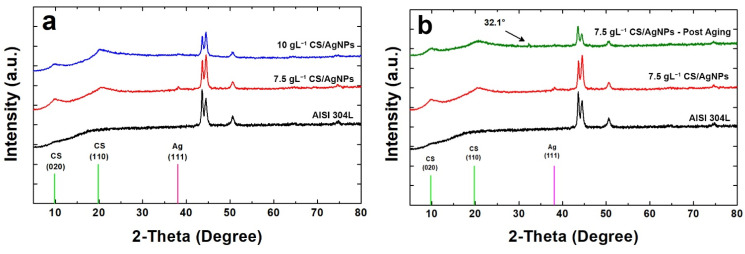
XRD patterns of the samples: (**a**) before and (**b**) after aging in SBF.

Coatings were also characterized by XRD analysis and diffraction patterns obtained are reported in Figure 4. The diffraction peaks of CS and silver were identified through comparisons with the ICDD database (card number 39-1894 for CS and 04-0783 for silver) [76]. For comparison, also the diffraction pattern of bare AISI 304L was reported, whose peaks were identified through the literature data [98]. In Figure 4a the characteristic peaks of both CS and silver can be observed in the coatings obtained using different concentrations of CS in the deposition bath. CS shows a semi-crystalline nature and in fact, according to the literature [99,100,101], two diffraction peaks are attributable to it. In particular, the first peak at 2θ = 9.6° (020) is relative to the hydrated structure and the second for 2θ equal to 19.8° (110) is due to the segments of the α-chitin chain.

The presence of silver was confirmed by the peak at 2θ equal to 38.07 (characteristic of (111) diffraction plane). This peak is of a very low intensity due to the low concentration of Ag, and also very wide as Ag is present as nanoparticles and therefore it is characterized by an extremely small average grain size. The secondary characteristic peak of silver at 2θ equal to 44.43° is not visible due to the overlap with the signal coming from the metal substrate that is predominant. In comparison with bare steel, in the samples the peaks of metallic substrate appear with less intensity, mainly for those obtained using a deposition solution of 10gL^−1^ CS. This is due to the presence of coating that shields the steel diffraction peaks.

In Figure 4b the diffraction pattern of the sample obtained using a solution with 7.5 gL^−1^ CS/AgNPs after 21 days of aging in SBF was reported. For a better comparison also the patterns of bare AISI 304L and the coating before aging was reported. The comparison highlights important results. The first concerns the disappearance of diffraction peak of the silver due to the release of AgNPs in SBF. This result is in line with EDS analysis as above reported. The second result is related to the appearance of the weak peak at about 2θ = 32.1° which is attributable to hydroxyapatite (HA) phase. The presence of HA phase on the surface of coating after the aging in SBF proves the bioactivity of this biopolymer [102,103]. In fact, according to Baskar et al., the polycationic nature of CS, due to amine group, induces phosphate ions adsorption leading to the nucleation of HA crystals [104]. Finally, as concern the crystallinity of CS, no change was observed before and after aging. In fact, the shape and intensity of the CS peaks is practically identical. Similar results were obtained for the sample 10 gL^−1^ CS/AgNPs after 21 days of aging in SBF (Figure S4).

**Figure 5:**
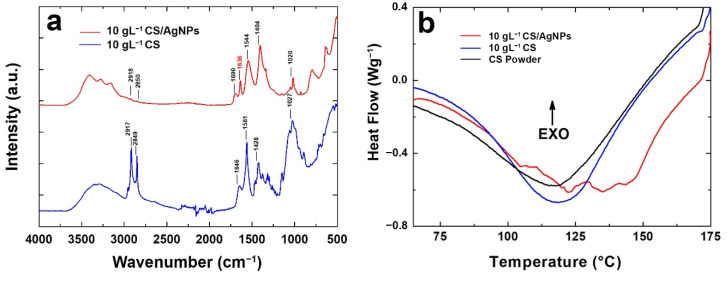
(**a**) FT-IR spectra and (**b**) DSC analysis of the coating with and without Ag nanoparticles.

FT-IR/ATR analysis was carried out and FT-IR/ATR spectra are shown in Figure 5a to compare coatings with and without AgNPs. According to the data reported in the literature [73,105,106], the interaction between AgNPs and CS can be studied via FT-IR/ATR spectra.

The broad peak of CS at 3330 cm^−1^ represents an overlap between O–H and N–H stretching vibrations the polysaccharide moieties [105]. For the CS/AgNPs sample, CS band relative to the hydroxyl groups shifted to a lower wavenumber and they are divided into three smaller bands likely due to the interaction of amide and amine groups with AgNPs [105]. In addition, the amide B bands in the CS/AgNPs spectrum, at 2850 cm^−1^, are smaller than pure CS, indicating the reduction in the hydrogen bond due to an interaction with AgNPs [106]. In addition, the characteristic shoulder peak of the amide-I (stretching), found at 1647 cm^−1^ in CS, shifts to 1636 cm^−1^ for CS/AgNPs films and it is reduced in intensity. This result was already observed in the scientific literature and related to an interaction between Ag, O and N atoms of these groups [73,105,107]. Similarly, at 1560 cm^−1^, CS shows the typical bending vibrations of amide-II that shifted to 1544 cm^−1^ for CS/AgNPs with a lower intensity, suggesting the chelating of AgNPs with CS [73,108,109]. In addition, the peak intensity reduction observed for CS/AgNPs in the range 1000 cm^−1^–1100 cm^−1^, ascribed to C–N stretching, confirms the complexation of AgNPs with CS, as reported elsewhere [108]. All these changes in the FTIR/ATR spectra of CS/AgNPs indicate the chelating of AgNPs with CS amino and hydroxyl groups.

Information about the thermal behavior of CS with and without AgNPs was obtained via DSC analysis, shown in Figure 5b. The endothermic peak of the pure chitosan coating was detected at about 120 °C. The same was detected for CS powder used as starting regent to prepare the deposition solution. A shift of the endothermic peak from 120 °C to 135 °C was observed for the coating containing the AgNPs. This phenomenon is typical of nanocomposite systems that are characterized by a higher melting temperature. In fact, nanoparticles tend to increase the crystallization of the polymer and they act as nucleation sites [110]. In addition, there was a slight increase in the melting enthalpy (Table S1) confirming the nucleating effect of AgNPs on CS [111,112,113].

The corrosion performances were evaluated in vitro through electrochemical characterizations carried out at 37 °C in SBF for different aging times. Before starting other corrosion tests, OCP monitoring was performed for 1 h (Figure S5). The monitoring of OCP is very useful to identify possible damage to the coatings. For the as-prepared samples (0 day of immersion), regardless of the chitosan concentration, a remarkable increase in OCP values of almost 80 mV during the test can be observed. This characteristic is attributable to the swelling of dehydrated CS/AgNPs samples that were dipped in SBF. Nevertheless, OCP curves of the samples collected during aging time are almost constant and higher with respect to bare steel. This behavior indicates a fairly stable coating that does not undergo substantial changes in SBF.

**Figure 6:**
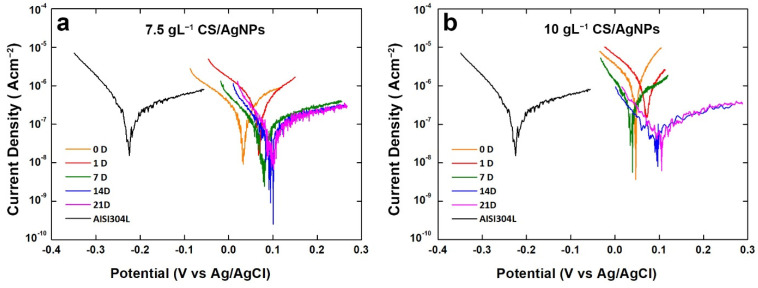
Polarization curves of the composite coatings obtained using different concentrations of CS after different aging times.

The results coming from polarization curves are in line with OCP monitoring (Figure 6). From potentiodynamic polarization the corrosion potential (E_corr_) and corrosion current density (i_corr_) were evaluated by extrapolation of Tafel curve, and the results are reported in Table 1. The value of E_corr_ increases with aging time, in line with OCP. With, respect to bare steel, coated samples have a positive value of E_corr_ just from the start of aging. In particular, a gain about 300 mV was measured, with respect to bare steel. During aging, E_corr_ increases up about 100 mV and it always remains above the corrosion potential of 304L steel.

Concerning i_corr_ values, it can be observed that at the end of aging both samples have a value of i_corr_ lower than bare steel. These good results are directly associated to the presence of coating that acts as a physical barrier slowing down the corrosion phenomena.

**Figure 7:**
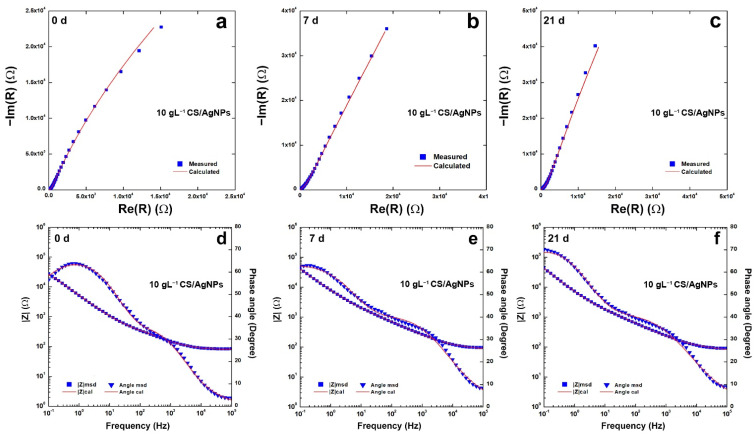
(**a**–**c**) Nyquist and (**d**–**f**) Bode plots of the composite coatings obtained using 10 gL^−1^ of CS after different aging time.

Impedance plots are reported in Figure 7 for the coating obtained using 10 gL^−1^ of CS. In the Nyquist plots, Figure 7a–c, the negative imaginary impedance is plotted versus the real part of the impedance. Figure 7d–f report the Bode plots where the logarithm of the total impedance is plotted versus the logarithm of the frequency. In these figures also, the variation with the frequency of the phase angle was reported. The change over time in of the impedance response agrees with the potentiodynamic polarization curves discussed above and with the behavior found in [82].

All impedance data were fitted with an equivalent circuit (EQ) (reported in Figure S6). The best fitting was obtained taking into account the equivalent circuit proposed in [114,115] that simulates the behavior of non-uniform coatings with the presence of defects. In particular, the equivalent circuit is R_s_(CPE_1_(R_1_(CPE_2_(R_2_W))), and was already used for modeling CS coatings [60]. In the equivalent circuit, Rs is the resistance of the solution, CPE_1_ and R_1_ simulate the behavior of the porous outer deposit layer in contact with the SBF solution, while CPE_2_ and R_2_ model the behavior of the layer in contact with the metal substrate. The Warburg element (W) was inserted to simulate the indicative processes within the porous structure. The use of CPE (constant phase element) has been used by Hinderliter to model non-homogeneous coating due to various geometries, presence of defects, and changes in composition [116,117]. As for the bare steel, a simpler circuit Rs(CPE_2_R_2_) was used taking into account the capacitance of double electrical layer and the resistances of the solution and the charge transport, respectively [116,117,118,119,120]. The values obtained from fitting (Table S2) were characterized by χ^2^ almost on the order of 10^−4^ and the relative error of each parameter is less than 10%. The presence of the Warburg element is an indication of the diffusive phenomena that occur inside the coating, since chitosan tends to swell when it is placed in SBF. Although the values of CPE_2_, n_2_ and R_2_ could be in the same order as the bare steel, the coating increases charge transport resistance in line with the results of Tafel extrapolation reported in Table 1.

**Figure 8:**
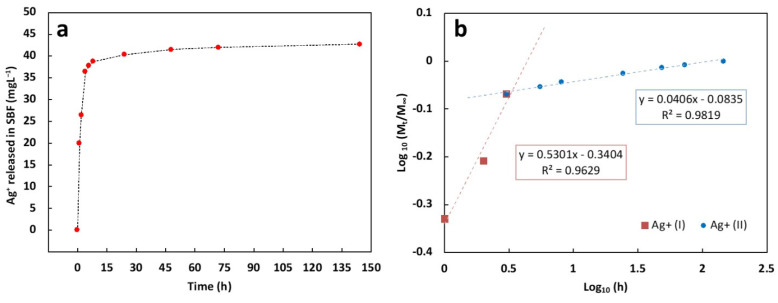
(**a**) Ag^+^ cumulative release curve from composite coating in SBF at 37 °C; (**b**) Log (*M_t_*/*M*_∞_) vs. Log (time) curves of the two release stages and their corresponding regression equations.

Ag^+^ release was evaluated by immersing an area of about 2 cm^2^ in 15 mL of SBF at 37 °C. As reported in Figure 8a, during the first three hours, a steep burst release can be highlighted, followed by sustained Ag^+^ release. In detail, by assuming the Ag^+^ released after 144 h as the total Ag^+^ released by the coating, during the first hour almost 50% and 85% of Ag^+^ was released after 1 and 3 h, respectively. After 48 h, nearly 97% of Ag^+^ was released and a plateau in the release curve was observed for the other time points. In order to investigate the release mechanism of the ions in SBF, the experimental data were fitted using the following power law model (Equation (9)):

(9) $$\frac{M_{t}}{M_{\infty }}=kt^{n}$$

*M*_∞_ represents the weight of Ag^+^ released after 144 h; *M_t_* is the weight of Ag^+^ released at the release time *t*; *k* is a kinetics constant; n is the diffusion exponent according to Peppas et al. [121]. In particular, if *n* < 0.5 the release is diffusion-controlled (Fickian) whereas it is defined swelling-controlled if *n* = 1.0. If 0.5 < *n* < 1.0, the release is controlled by the superposition of both phenomena and it is defined as anomalous transport [121].

According to our previous work [122], the power law model was used to fit separately two release stages (coded as Ag^+^ (I) and Ag^+^ (II)) that can be distinguished by the linearity of the Log (*M_t_*/*M*_∞_) versus Log (time) curves, reported in Figure 8b together with their corresponding regression equations. The n value of the burst release (*t* ≤ 3 h, *n* ∼ 0.53) and the sustained release (*t* ≥ 3 h, *n* ∼ 0.04), revealed that the release mechanism is slightly anomalous during the first three hours while it switches to a diffusion release from the third hour until the end of the test, according to other works [123].

## 4. Conclusions

In this work, CS/AgNPs composite coating on AISI 304L was obtained through galvanic deposition. CS/AgNPs composite coating were obtained using a solution of CS at different composition. The formation of the coating occurs due to the electrogeneration of base reactions that lead to the increase in pH at the electrode/electrolyte interface. This pH increase ensures the precipitation of CS on the surface of electrode with the concomitant incorporation of Ag nanoparticles present in the deposition solution. SEM images revealed that coatings are able to cover the entire surface of the substrate. In addition, the presence of AgNPs in composite was shown. The presence of AgNPs was also confirmed by EDS analysis where Ag peaks were found. X-ray diffraction patterns presented characteristics of composite coating in which diffraction peaks of CS and Ag peaks were identified. After an aging period, the XRD and EDS results attested to a decrease in AgNPs due to the release and the appearance of the HA phase. No change in the crystallinity of CS was observed. SEM analysis reveals that after aging, the morphology of the coating was practically unchanged. This result is imputable to the stability of the coating in the physiological conditions. According to the XRD results post aging, the EDS tests reveal the presence of new elements due to the HA formation in SBF. This result is very interesting because suggests that CS/AgNPs are bioactive. Using FT-IR and DSC analyses, the differences between composite coating and pure CS were studied. AgNPs interact with polymeric matrix causing a shift of a typical vibration mode of CS in the FT-IR spectra. As regards DSC analysis, an increase in melting temperature in composite coating samples was observed due to the presence of the nanoparticles. Corrosion tests were carried out for 21 days in SBF at 37 °C and the results proved that corrosion potential values kept higher than bare steel and concurrently increased with aging time. A lower corrosion current density with respect to uncoated AISI 304L was also measured. Hence, coatings performances testify good protection from corrosion phenomena. In fact, EIS analysis results have shoved an increasing of charge transport resistance for the coated sample. Furthermore, the coating showed an Ag^+^ release in SBF at 37 °C up to 144 h, characterized by an initial burst release for the first three hours, followed by a sustained release with a diffusion-controlled mechanism.

## Figures and Tables

**Table 1:** Corrosion potential (E_corr_) and corrosion current density (i_corr_) extrapolated by Tafel curve for the coatings after different aging time in SBF.

	Time (day)					
	0	1	7	14	21	AISI 304L
**7.5gL^−1^ CS/AgNPs**						
E_corr_ (mV)	30	69	78	81	94	−225
i_corr_ (Acm^−2^)	7.02 × 10^−7^	5.52 × 10^−7^	7.78 × 10^−8^	8.52 × 10^−8^	9.06 × 10^−8^	2.47 × 10^−7^
**10gL^−1^** **CS/AgNPs**						
E_corr_ (mV)	46	63	35	85	91	−225
i_corr_ (Acm^−2^)	2.89 × 10^−6^	5.23 × 10^−7^	4.22 × 10^−7^	6.24 × 10^−8^	7.32 × 10^−8^	2.47 × 10^−7^

## Data Availability

Not applicable.

## Supplementary Materials

The following supporting information can be downloaded at: https://www.mdpi.com/article/10.3390/polym14183915/s1, Figure S1: calibration line of silver sulfadiazine in SBF obtained via UV-VIS absorbance spectra at 262 nm; Figure S2: (a) UV-VIS spectra of cathodic solution. (b) Solution with CS and (c) CS/AgNPs; Figure S3: (a) SEM image of coating and (b) relative EDS spectrum of the coating obtained using a solution of 10gL^−1^ of CS after immersion in SBF for 21 days; Table S1: melting enthalpy calculate by DSC; Figure S4: XRD patterns of the sample 10 gL^−1^ CS/AgNPs after aging in SBF; Figure S5: OCP curves after different aging time: (a) coating obtained using a solution of 7.5 gL^−1^ of CS, (b) coating obtained using a solution of 10gL^−1^ of CS.; Figure S6: schematic cross-section of the coating and equivalent circuit used to fitting the impedance data; Table S2: fitting parameters of impedance data of the sample 10 gL^−1^ CS/AgNPs. For comparison, the value of uncoated AISI 304L fitted with an R(CPE(R)) equivalent circuit was also reported. The mean standard deviation was 3.7%.
