# Supplementary material for: Composite Coatings of Chitosan and Silver Nanoparticles Obtained by Galvanic Deposition for Orthopedic Implants
Source: Polymers (Basel). 2022 Sep 19;14(18):3915. doi: 10.3390/polym14183915 (PMC9504697; doi:10.3390/polym14183915)
Supplement: Supplementary file 1 [file polymers-14-03915-s001.zip › polymers-1914072-supplementary.pdf]

# Composite Coatings of Chitosan and Silver Nanoparticles Obtained by Galvanic Deposition for Orthopedic Implants

**C. Zanca [1], S. Carbone [1], B. Patella [1], F. Lopresti [1], G. Aiello [1], V. Brucato [1], F. Carfì Pavia [1,2], V. La Carrubba [1,3] and R. Inguanta [1,*]**

[1] Department of Engineering, University of Palermo, RU INSTM, Viale delle Scienze, 90133 Palermo, Italy
[2] Consorzio Universitario di Caltanissetta, Corso Vittorio Emanuele 92, 93100 Caltanissetta, Italy
[3] ATeN Center, University of Palermo, Viale delle Scienze, 90133 Palermo, Italy
* Correspondence: rosalinda.inguanta@unipa.it

**Supplementary**

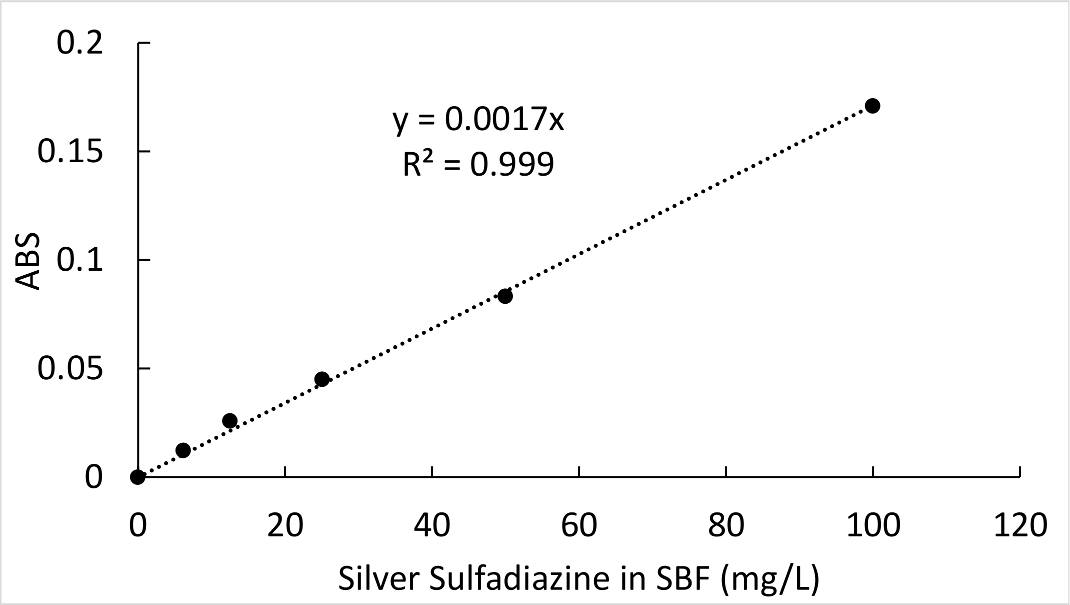


**Figure S1.** Calibration line of Silver Sulfadiazine in SBF obtained via UV-Vis absorbance spectra at 262 nm

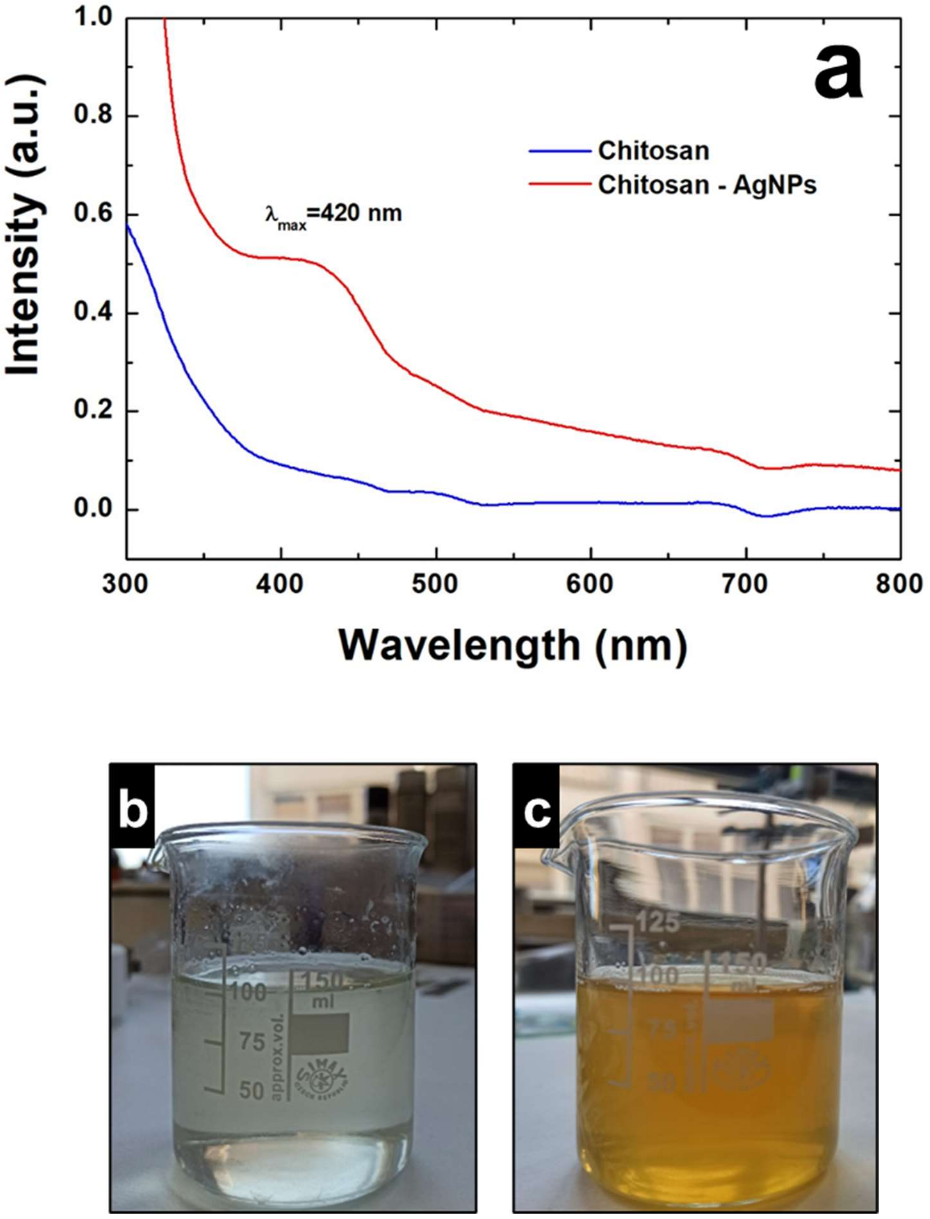


**Figure S2.** (a) UV-VIS spectra of cathodic solution. (b) Solution with CS and (c) CS/AgNPs

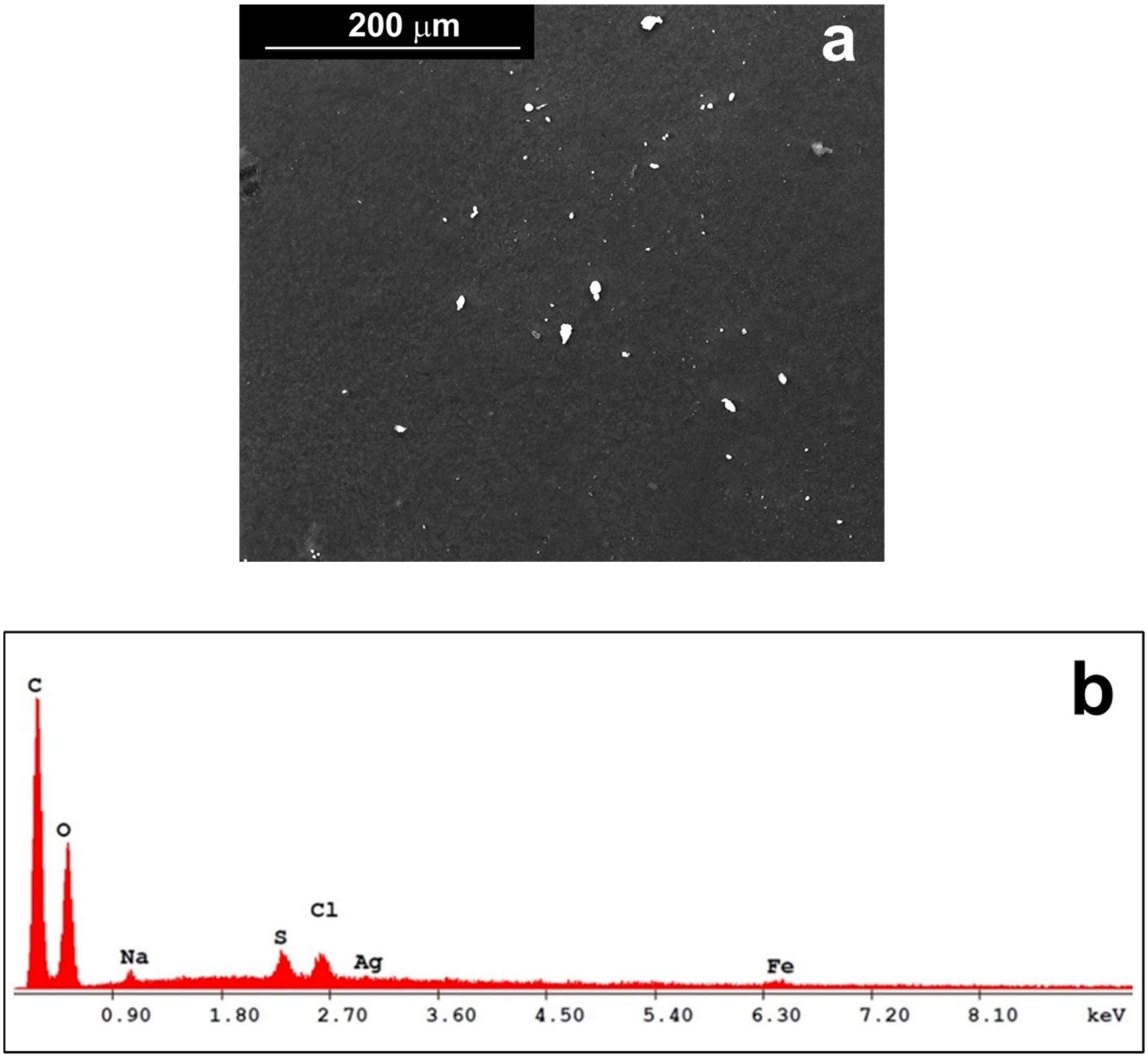


**Figure S3** (a) SEM image of coating and (b) relative EDS spectrum of the coating obtained using a solution of $10 gL^{-1}$ of CS after immersion in SBF for 21 days.

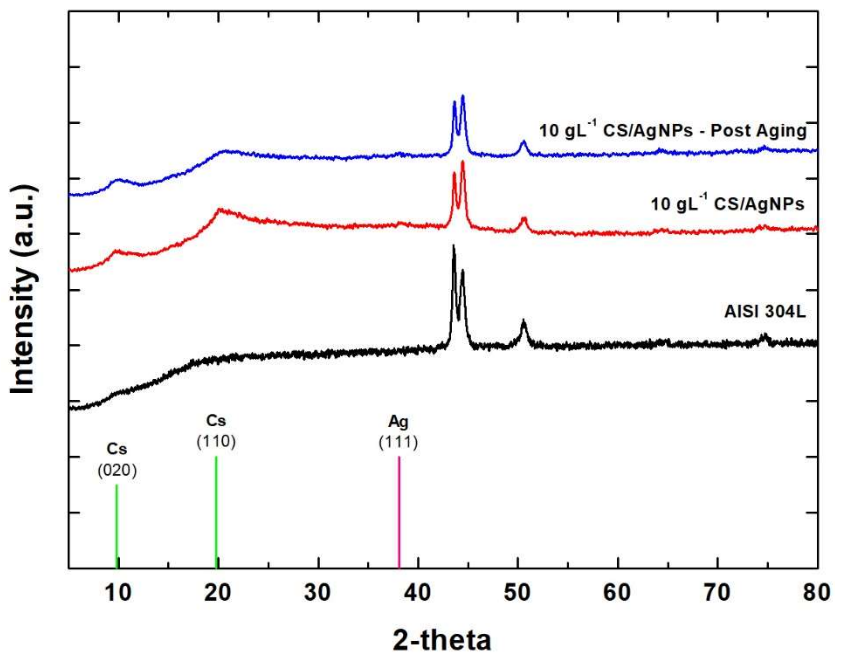


**Figure S4.** XRD patterns of the sample 10 $gL^{-1}$ CS/AgNPs after aging in SBF.

| | ΔH [J/g] |
|---|---|
| **Chitosan powder LMW** | **252** |
| **10 $gL^{-1}$ CS** | **213** |
| **10 $gL^{-1}$ CS/AgNPs** | **237** |

**Table S1.** Melting enthalpy calculate by DSC

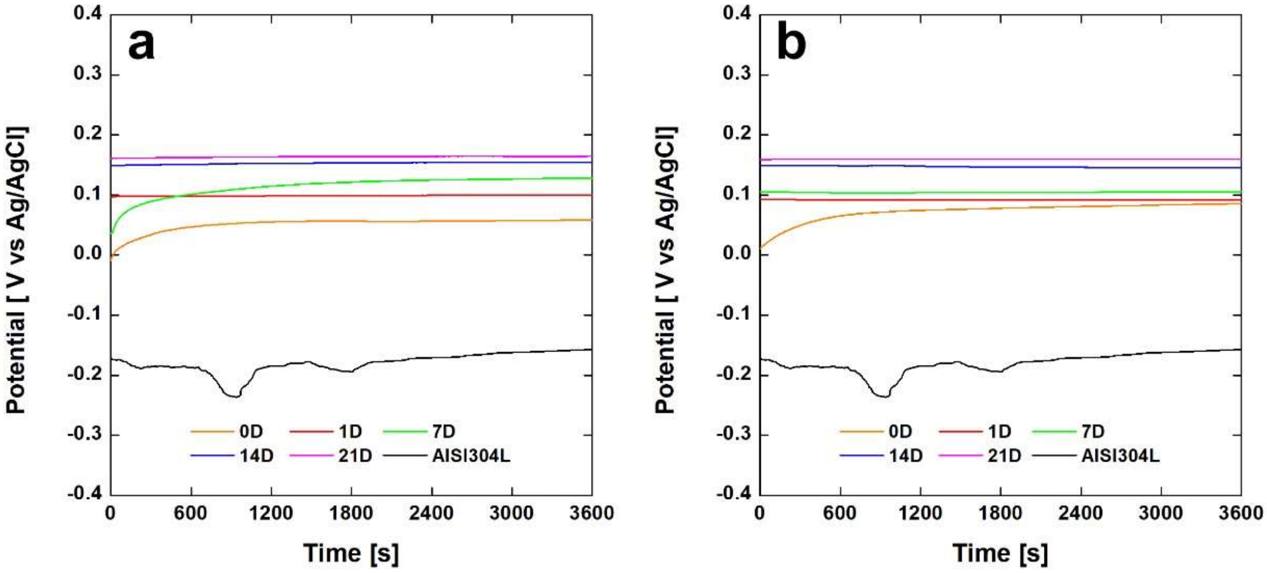


**Figure S5** OCP curves after different aging time: a) coating obtained using a solution of 7.5 $gL^{-1}$ of CS, b) coating obtained using a solution of 10$gL^{-1}$ of CS.

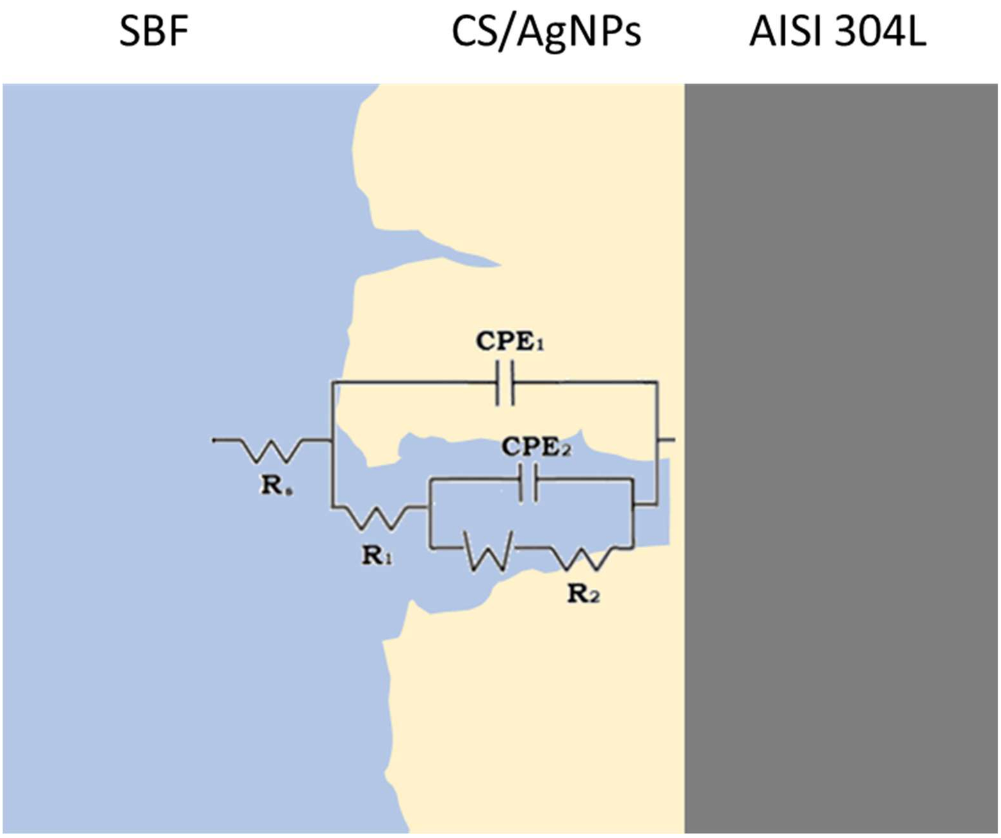


**Figure S6** Schematic cross-section of the coating and equivalent circuit used to fitting the impedance data

| | Time (Day) | | | | | |
|---|---|---|---|---|---|---|
| | 0 | 1 | 7 | 14 | 21 | AISI 304L |
| | R(CPE1(R(CPE2(RW)))) | | | | | R(CPE R) |
| $R_s$ | 77.81 | 82.89 | 79.26 | 75.49 | 73.42 | 32.62 |
| $CPE_1$-Y | $4.59\times10^5$ | $3.09\times10^5$ | $5.04\times10^5$ | $6.19\times10^5$ | $6.16\times10^5$ | - |
| $CPE_1$-$n_1$ | 0.6489 | 0.653 | 0.5419 | 0.5076 | 0.5218 | - |
| $R_1$ | 237.4 | 564.2 | 1053 | 1728 | 1612 | - |
| $CPE_2$-Y | $4.77\times10^5$ | $4.06\times10^5$ | $3.23\times10^5$ | $3.3\times10^5$ | $3.41\times10^5$ | $5.51\times10^5$ |
| $CPE_2$-$n_2$ | 0.7602 | 0.7297 | 0.7465 | 0.7953 | 0.8123 | 0.7665 |
| $R_2$ | $1.14\times10^5$ | $4.97\times10^5$ | $4.48\times10^4$ | $1.55\times10^6$ | $9.21\times10^5$ | $5.38\times10^5$ |
| W | $4.36\times10^5$ | $2.61\times10^5$ | $4.11\times10^6$ | $2.85\times10^5$ | $1.98\times10^5$ | - |

**Table S2** Fitting parameters of impedance data of the sample 10 gL-1 CS/AgNPs. For comparison, the value of uncoated AISI 304L fitted with an R(CPE(R)) equivalent circuit was also reported. The mean standard deviation was 3.7%
